# Role of non-invasive methods in detecting liver impairment in familial Mediterranean fever adult patients with persistent hepatic cytolysis

**DOI:** 10.1038/s41598-022-17358-x

**Published:** 2022-10-05

**Authors:** Samuel Deshayes, Thibault Fraisse, Soraya Fellahi, Olivier Steichen, Léa Savey, Bruno Turlin, Mona Munteanu, Achille Aouba, Rim Bourguiba, Véronique Hentgen, Jean-Manuel Faintuch, Irina Giurgea, Gilles Grateau, Jean-Philippe Bastard, Sophie Georgin-Lavialle

**Affiliations:** 1 GRAASU GRC, Service de Médecine Interne, Centre de Référence des Maladies Autoinflammatoires et des Amyloses Inflammatoires (CEREMAIA), FHU PACEMM, INSERM U938, Sorbonne Université, Hôpital Tenon, AP-HP, 75020 Paris, France; 2 Service de Médecine Interne, UNICAEN, UR4650 PSIR, Normandie Univ, CHU de Caen Normandie, 14000 Caen, France; 3 GRAASU GRC, INSERM UMR_S 938, Centre de Recherche Saint-Antoine (CRSA), RHU CARMMA, Institute of Cardiometabolism and Nutrition (ICAN), Sorbonne Université, 75012 Paris, France; 4 Département de Biochimie-Pharmacologie-Biologie Moléculaire-Génétique Médicale, Hôpitaux Universitaires Henri Mondor, AP-HP, Créteil, France; 5 Laboratoire d’Anatomie Pathologique, CHU Rennes, Rennes, France; 6 Department of Hepatology, Pitié-Salpêtrière Hospital, AP-HP, Paris, France; 7 Service de Pédiatrie Générale, CEREMAIA, CH de Versailles, Le Chesnay, France; 8 Service d’Imagerie Médicale, Sorbonne Université, Hôpital Tenon, AP-HP, 75020 Paris, France; 9 Laboratoire de Génétique, Sorbonne Université, Hôpital Trousseau, AP-HP, 75012 Paris, France; 10 Institut National de la Santé et de la Recherche Médicale (INSERM) U955, RHU CARMMA, Institut Mondor de Recherche Biomédicale (IMRB), Université Paris Est Créteil, 94010 Créteil, France

**Keywords:** Innate immunity, Clinical genetics

## Abstract

Familial Mediterranean fever (FMF) patients may have hepatic cytolysis, although its origin is not formally elucidated. We aimed to evaluate liver involvement in familial Mediterranean fever (FMF) using non-invasive methods. All adult FMF patients harboring two non-ambiguous mutations of the MEFV gene with hepatic cytolysis were identified in a French tertiary adult center for FMF. Liver impairment was explored with FibroMax (a non-invasive method to estimate hepatic steatosis, necrosis, inflammation and fibrosis) and liver ultrasound. Among 520 FMF adult patients, 43 had persistent hepatic cytolysis and 20 patients were included (11 women, median age at inclusion: 49.5 years). According to the FibroMax results, patients were classified as having steatosis, fibrosis, and possible or definite nonalcoholic steato-hepatitis in 10 (50%), 9 (45%) and 7 (35%) of cases, respectively. The score of steatosis did not seem associated with the usual metabolic risk factors. No significant association was found between the cumulated dose of colchicine and any of the scores included in FibroMax. In adult FMF patients with persistent hepatic cytolysis, steatosis is the first cause to consider even in the absence of usual metabolic risk factors, suggesting other mechanisms. Colchicine did not seem to be involved in this toxicity.

## Introduction

Familial Mediterranean Fever (FMF) is the most frequent monogenic auto-inflammatory disease, secondary to mutations in the *MEFV* gene. Patients usually originate from the Mediterranean area and have recurrent self-limited episodes of fever accompanied by abdominal, chest and/or joint pain. Daily treatment with colchicine reduces the risks of crisis and inflammatory AA amyloidosis^1^.

Some FMF patients exhibit abnormal liver tests, but it is not clear whether these abnormalities are secondary to acute or chronic inflammation, FMF treatment, AA amyloidosis, or comorbidities. Only few small case series have focused on these patients, and found a probable association of FMF with nonalcoholic fatty liver disease (NAFLD) and cryptogenic cirrhosis^2^. However, the prevalence of obesity and type II diabetes or impaired fasting glycemia, two major risk factors for NAFLD^3^, among FMF patients with NAFLD is lower than in NAFLD patients without FMF^4^. These data suggest that recurrent or chronic inflammation, through pro-inflammatory cytokines including interleukin-1, may promote NAFLD in FMF patients^2^.

The aim of this study was to evaluate liver involvement in a cohort of adult FMF patients using non-invasive methods with persistent elevated transaminase levels, and to correlate their results to the presence of metabolic syndrome and treatment with colchicine.

## Methods

### Patients

All adult patients followed in the French national reference center for FMF who previously had elevated transaminase levels (aspartate aminotransferase ASAT > 32 UI/L and/or alanine aminotransferase ALAT > 43 UI/L) checked twice in the two preceding years were contacted for clinical examination and blood tests along with liver ultrasound to explore their liver abnormalities. We excluded patients with biopsy-proven AA amyloidosis or with impaired renal function and proteinuria, as well as patients who did not fulfill Livneh FMF criteria^5^ or who did not harbor two validated pathogenic *MEFV* mutations as defined in the Infevers online registry (https://infevers.umai-montpellier.fr/web/). We also excluded those with a daily alcohol consumption above 30 g per day or who had another cause of elevated liver tests (see below). During clinical examination, the height was precisely measured without shoes by a stadiometer and the weight without clothes by a scale to the nearest kilogram. Waist circumference was then evaluated from mid-point between the navel and the tenth rib. Body mass index (BMI) was calculated by dividing the weight in kg by the height squared (m^2^). Patients were questioned about hypertension or the use of antihypertensive, antidiabetic or antilipidemic drugs. Metabolic syndrome in its latest definition^6^ was diagnosed if the patient fulfilled at least 3 of the following criteria: elevated waist circumference adjusted in the concerned population, elevated triglyceride (TG) level above 150 mg/dL, elevated fasting glycemia above 100 mg/L, reduced high-density lipoprotein (HDL)-cholesterol level less than 40 mg/dL in men or less than 50 mg/dL in women, any treatment for hypertension along with a history of hypertension. No patient was in crisis on the day of the assessment.

### Biological parameters

We excluded another cause for elevated liver tests by looking for: hepatitis B; hepatitis C; Epstein-Barr virus; cytomegalovirus; creatine phosphokinase; alpha 1 anti-trypsin; ceruloplasmin; ferritin; transferrin saturation coefficient; anti-transglutaminase and -endomysium antibodies; anti-nuclear antibodies; anti-smooth muscle antibodies and anti-liver-kidney microsomal antibodies.

After overnight fasting, blood samples were taken and analyzed for the following biological parameters: ASAT; ALAT; gamma-glutamyl-transpeptidase (GGT); bilirubin; alkaline phosphatase (ALP); glycemia; glycated hemoglobin (HbA1c); total cholesterol; HDL; low-density lipoprotein (LDL); TG; insulin; high-sensitivity C-reactive protein (hsCRP) and serum amyloid A (SAA).

hsCRP and SAA were measured by nephelometry on an IMMAGE analyzer (Beckman-Coulter, Villepinte, France). Insulinemia was assayed by chemiluminescence (ARCHITECT Insulin Abbott, Rungis, France). ASAT, ALAT, GGT, bilirubin, glucose, total cholesterol, HDL, LDL and TG levels were routinely assayed on ARCHITECT Ci8200 (Abbott, Rungis, France). HbA1c was performed on CAPILLARYS (SEBIA, Lisses, France).

We also used the FibroMax (Biopredictive, Paris, France), following the recommended pre‐analytical and analytical conditions, that combines different validated scores in NAFLD to non-invasively estimate liver involvement, in particular hepatic steatosis (SteatoTest), necrosis, inflammation (NashTest) and fibrosis (FibroTest) at the same time^7,8^. It relies on the combination of the following parameters: serum α2-macroglobulin, apolipoprotein A1, haptoglobin, total bilirubin, GGT, ALAT, ASAT, total cholesterol, TG, fasting glucose, BMI, age and sex. The SteatoTest can quantitatively assess liver steatosis and the score estimates liver steatosis percentage according to the following scale: score less than 0.37: S0 (no steatosis); score between 0.38 and 0.56: S1 (1 to 5% of steatosis); score between 0.57 and 0.68: S2 (6 to 32% of steatosis); score above 0.69: S3 (> 32% of steatosis)^9,10^. We decided to combine S1 and S2 categories. The FibroTest provided a quantitative estimation of liver fibrosis, and the score can estimate the hepatic fibrosis according to the METAVIR scoring system: score less than 0.27: F0 or F0–F1 (no fibrosis), score between 0.27 and 0.48: F1 or F1–F2 (minimal fibrosis), score between 0.48 and 0.58: F2 (moderate fibrosis), score between 0.58 and 0.74: F3 or F3–F4 (advanced fibrosis), score above 0.74: F4 (severe fibrosis)^11^. We decided to combine the F1 or F1–F2 and F2 categories, and the F3 or F3–F4 and F4 categories. The NashTest has been validated in the detection of NASH among NAFLD patients^7,12^. Results are expressed as: score at 0.25: N0 (absence of NASH); score at 0.5: N1 (possible NASH); score at 0.75: N2 (NASH)^12^. We decided to combine N1 and N2 categories.

### Liver ultrasound

Patients had a liver ultrasound performed by experienced radiologists who were blinded to the clinical and biological status of the patients. In order to improve the interpretation of liver ultrasound, results were given as presence or absence of steatosis. All examinations were made with a Hitachi ARIETTA V70A ultrasound machine first used in January 2016. We chose this type of imaging because of its accessibility and its low cost with a reasonable reliability in detecting steatosis^13^.

### MEFV sequencing

All patients had *MEFV* exon 10 sequencing by Sanger technique as previously described^14^.

### Ethics

The study was conducted in accordance with the recommendations of the Declaration of Helsinki. Patients were included in the JIR-cohort, an international multicenter data repository established by the National Commission on Informatics and Liberty (CNIL authorization number N°: 914677), and were informed that data collected in medical records might be used for research study in accordance with privacy rules. The experimental protocols were approved by the IRB (CECIC Rhône-Alpes-Auvergne, Clermont-Ferrand, IRB 5891, n°2014-04) and the written consent was waived for the study. The work-up performed for each patient was part of routine care, due to the presence of liver abnormalities.

### Statistical analyses

Continuous variables were reported as medians [quartile 1–quartile 3] and analyzed using the nonparametric Mann–Whitney test when two groups were compared, or the non-parametric Kruskal–Wallis test when more than two groups were compared. Categorical variables were expressed as percentages. The Spearman correlation coefficient was calculated to determine correlations between two continuous variables, and the R package “corrplot” was used to visualize the correlation matrix^15^. Associations were considered significant if the *p* value was < 0.05 and the q-value (i.e., the false discovery rate using the Benjamini–Hochberg correction method) was < 0.1. Statistical analyses were done using R 4.0.4.^16^.

## Results

### Main features of the patients

In our cohort of 520 FMF adult patients followed in the French national reference center for FMF, 234 patients previously had liver biochemical tests. Among them, 43 with elevated transaminase levels checked twice in the two preceding years were contacted for clinical examination and blood tests along with liver ultrasound to explore their liver abnormalities. Overall, 20 adult FMF patients agreed to participate and were included to have all the assessments, consisting in 11 women and 9 men with a median age of 49.5 [29.75–56.25] years (Table 1), and with 5 patients (25% of the cohort) who no longer had an elevated ASAT and ALAT levels. Eighteen patients (90%) were homozygous for the M694V mutation of the *MEFV* gene and 2 were compound heterozygous for the M694V/V726A mutation (Table 2). All of them received colchicine for a median duration of 24.5 [17.25–36] years with a median cumulative dose of 15.5 [9.85–23.42] g and at a current median daily dose of 1.75 [1.37–2] mg. In addition, two of them were also treated by anti-IL-1 therapy. The median BMI was 23.75 [22.3–25.77] kg/m^2^ and the median waist circumference was 78 [72–97.5] cm (data available for 19 patients). All but 2 patients had liver ultrasound, and was normal in 10 (55%) patients, found hepatomegaly in 4 (22%) and steatosis in the remaining 4 (22%) patients.

**Table 1:** Main clinical and biological features of the included familial Mediterranean fever patients.

	n (%) or median [Q1–Q3] (n available)
**Clinical characteristics**	
Female, n (%)	11 (55)
Age (years), median [Q1–Q3]	49.5 [29.75–56.25]
BMI (kg/m^2^), median [Q1–Q3]	23.75 [22.3–25.77]
Waist circumference (cm), median [Q1–Q3]	78 [72–97.5] (n = 19)
Cumulative dose of colchicine (g), median [Q1–Q3]	15.5 [9.85–23.42]
Previously diagnosed diabetes, n (%)	0
High blood pressure, n (%)	4 (20)
Metabolic syndrome, n (%)	5 (25)
**Biological characteristics**	
Creatininemia (µmol/L), median [Q1–Q3]	77 [61–85]
C-reactive protein (mg/L), median [Q1–Q3]	2.82 [1.04–4.34]
Serum amyloid A (mg/L), median [Q1–Q3]	0 [0–7.425]
Fasting glycemia (mmol/L), median [Q1–Q3]	4.96 [4.66–5.49]
Glycated hemoglobin (%), median [Q1–Q3]	5.2 [5–5.4]
Aspartate aminotransferase (UI/L), median [Q1–Q3]	34 [29.25–48]
Alanine aminotransferase (UI/L), median [Q1–Q3]	53 [38.5–65]
Gamma-glutamyl-transpeptidase (UI/L), median [Q1–Q3]	29.5 [22–79.5]
Total bilirubin (µmol/L), median [Q1–Q3]	8 [7–12]
Alkaline phosphatase (UI/L), median [Q1–Q3]	79 [66.25–95.25]
High-density lipoprotein cholesterol (mmol/L), median [Q1–Q3]	0.91 [0.82–1.15]
Low-density lipoprotein cholesterol (mmol/L), median [Q1–Q3]	2.59 [1.84–3.11]
Triglyceride (mmol/L), median [Q1–Q3]	1.21 [0.89–1.52]
Insulin (pmol/L), median [Q1–Q3]	66.05 [56.32–111.55]
**FibroMax**	
SteatoTest score, median [Q1–Q3]	0.30 [0.22–0.51]
FibroTest score, median [Q1–Q3]	0.25 [0.13–0.43]
NashTest score, median [Q1–Q3]	0.25 [0.25–0.50]

Values are displayed as absolute number (%) or as median [quartile 1–quartile 3].

**Table 2:** Clinical and biological characteristics of each included familial Mediterranean fever patient.

Patient (age in years, sex)	Mutations	ASAT/ALAT-GGT/ALP	SteatoTest	FibroTest	NashTest	Liver ultrasound
Patient 1 (56, W)	M694V/M694V	27/34–31/63	S0	F0–F1	N0	Normal
Patient 2 (46, M)	M694V/M694V	43/82–30/78	S1–S2	F1–F2	N1	Normal
Patient 3 (70, W)	M694V/M694V	65/68–77/75	S3	F4	N2	Steatosis
Patient 4 (57, W)	M694V/M694V	35/33–12/67	S0	F0–F1	N0	Normal
Patient 5 (68, W)	M694V/M694V	51/49–41/90	S1–S2	F1–F2	N1	Hepatomegaly
Patient 6 (24, M)	M694V/M694V	33/53–24/64	S0	F1–F2	N0	Not done
Patient 7 (34, W)	M694V/M694V	14/19–13/88	S0	F0	N0	Hepatomegaly
Patient 8 (56, W)	M694V/M694V	62/64–92/93	S1–S2	F1–F2	N0	Normal
Patient 9 (56, M)	M694V/M694V	39/64–87/94	S3	F3	N1	Steatosis
Patient 10 (30, W)	M694V/M694V	23/21–16/59	S0	F0	N0	Hepatomegaly
Patient 11 (56, M)	M694V/M694V	48/92–93/126	S3	F3	N1	Steatosis
Patient 12 (29, M)	M694V/M694V	80/152–89/80	S3	F0	N1	Steatosis
Patient 13 (69, M)	M694V/M694V	46/45–93/116	S0–S1	F4	N0	Normal
Patient 14 (53, M)	M694V/V726A	25/60–12/62	S0	F1–F2	N0	Normal
Patient 15 (31, M)	M694V/M694V	32/63–29/99	S0–S1	F0	N0	Normal
Patient 16 (21, W)	M694V/V726A	30/73–28/77	S0	F0–F1	N0	Normal
Patient 17 (57, W)	M694V/M694V	32/40–28/69	S0	F0	N0	Normal
Patient 18 (26, W)	M694V/M694V	48/53–29/61.5	S0	F0	N0	Normal
Patient 19 (31, M)	M694V/M694V	26/50–41/131	S1–S2	F0	N1	Hepatomegaly
Patient 20 (21, W)	M694V/M694V	31/34–13/122	S0	F0	N0	Not done

*M* Man, *W* Woman, *ASAT* Aspartate aminotransferase, *ALAT* Alanine aminotransferase, *GGT* Gamma-glutamyl-transpeptidase, *ALP* Alkaline phosphatase.

### Liver test abnormalities

GGT, ALP and total bilirubin levels were moderately elevated (< 3 times the upper limit of normal) in 8 (40%), 4 (20%) and 0 patients, respectively, with median values of 29.5 [22–79.5] UI/L (normal values < 32), 79 [66.25–95.25] UI/L (normal values < 115) and 8 [7–12] µmol/L (normal values <  17), respectively. Eleven patients (55%) still had elevated ASAT levels with a median value of 34 [29.5–48] UI/L, and 14 (70%) still had elevated ALAT levels with a median value of 53 [38.5–65] UI/L. The search for differential diagnosis of liver involvement (see Methods) was negative in all patients. Of note, patients who no longer had an elevated ASAT and ALAT levels (n = 5, 25%) had normal FibroMax results. Only the 4 patients with a SteatoTest above 0.69 had steatosis on liver ultrasound, and 3 of them had a FibroTest above 0.58 (advanced to severe fibrosis).

### Correlations

**Figure 1:**
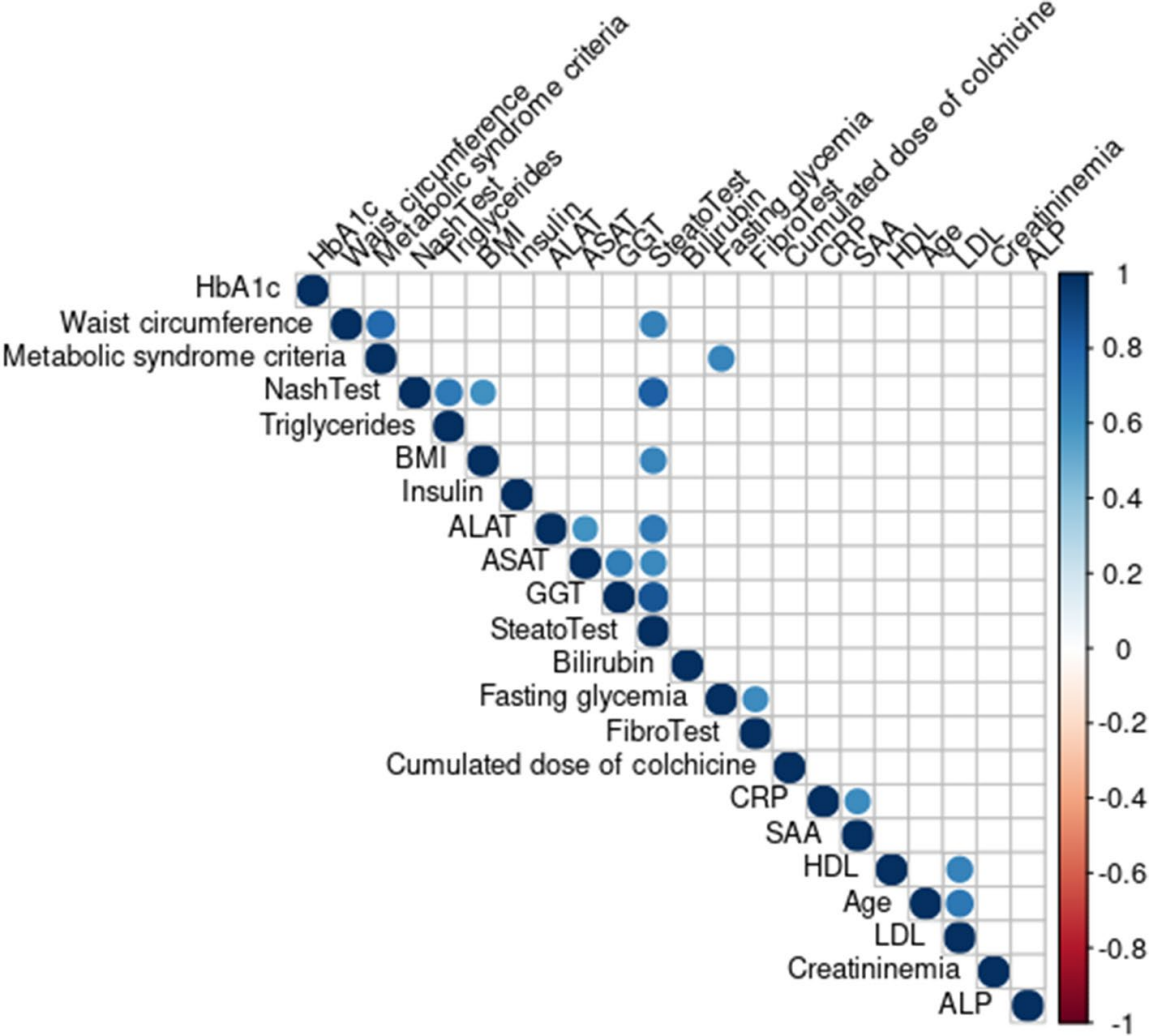
Correlations between clinical and biological parameters. The size and color intensity of the circles are proportional to the correlation coefficient, calculated with the nonparametric Spearman correlation test. Only statistically significant correlations (*p* < 0.05 and q-value, i.e., the false discovery rate using the Benjamini–Hochberg correction was < 0.1) are indicated. *ALAT* Alanine aminotransferase, *ASAT* aspartate aminotransferase, *GGT* Gamma-glutamyl-transpeptidase, *HbA1c* Glycated hemoglobin, *BMI* Body mass index, *CRP* C-reactive protein, *SAA* Serum amyloid A, *LDL* Low-density lipoprotein, *HDL* High-density lipoprotein, *ALP* Alkaline phosphatase.

Correlations between clinical and biological parameters are depicted on Fig. 1. The cumulated colchicine dose was not significantly correlated to any other parameter. As expected, each score was mainly correlated to its components. Therefore, the score at the SteatoTest was significantly correlated with ALAT, ASAT, GGT levels, waist circumference, BMI and results at the NashTest. The score at the NashTest was significantly correlated to the results at the SteatoTest, BMI and triglyceridemia. The score at the FibroTest was only correlated to fasting glycemia.

### FibroMax

In addition to significant differences between the parameters included in the calculation of SteatoTest as expected (i.e., GGT, ALAT, TG, BMI), there were significant differences in the number of criteria for metabolic syndrome, waist circumferences, glycated hemoglobin, alkaline phosphatase and NashTest according to the results of the SteatoTest (Supplementary Table 1). Ten patients (50%) were classified as having steatosis (S1 to S3), including 4 (20%) with a score suggestive of severe steatosis (S3).

**Figure 2:**
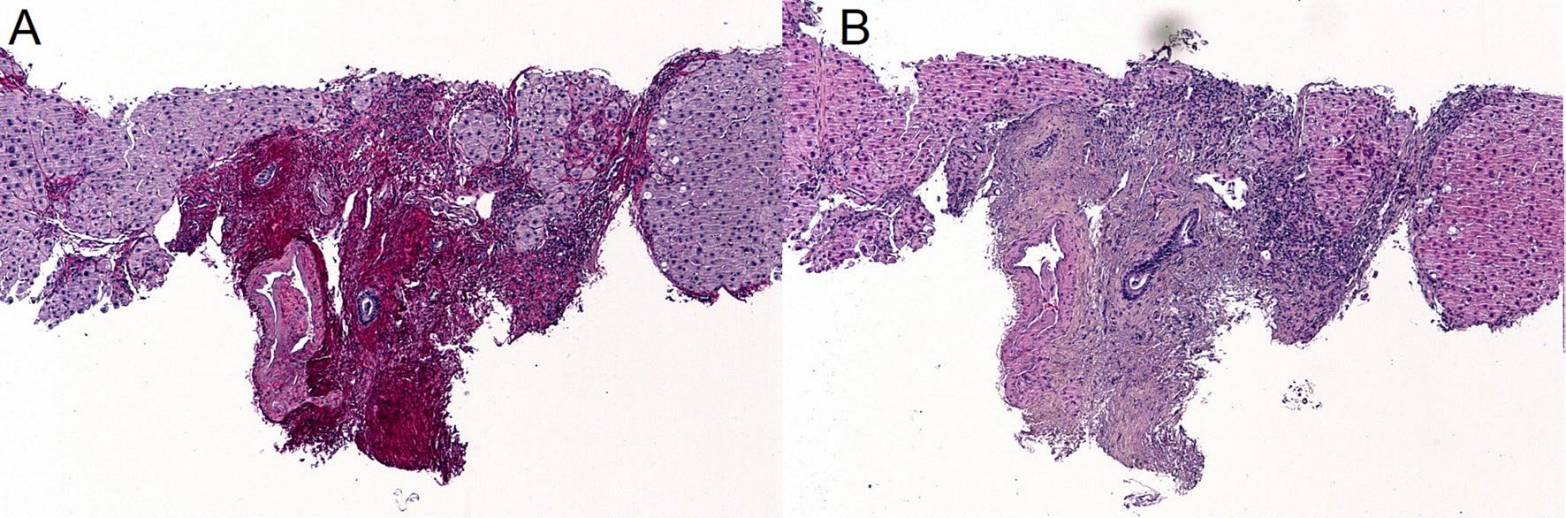
Histological appearance of liver biopsies from a patient suffering from familial Mediterranean fever with a FibroTest score of F4, suggestive of severe fibrosis. Fibrous septa seen on Sirius red (**A**) and Hematoxylin–eosin-saffron (**B**) histological sections, magnification × 5, confirming liver cirrhosis.

Regarding FibroTest (Supplementary Table 2), no significant differences were observed after adjustment for multiple comparisons. Nine patients (45%) were classified as having fibrosis (F1 to F4), including 4 (20%) with a score suggestive of advanced to severe fibrosis (F3 and F4). Results of the liver biopsies performed in a patient with a score suggestive of severe fibrosis (F4) are shown in the Fig. 2.

For NashTest (Supplementary Table 3), in addition to significant differences between the parameters included in the calculation of this score (i.e., GGT, BMI, TG), significant differences were observed between ALAT and SteatoTest according to the results of the NashTest. Seven patients (35%) were classified as having possible or definite NASH (N1 or N2).

Of note, no significant association was found between the cumulated dose of colchicine and any of these 3 scores.

## Discussion

In this study of 20 adult FMF patients with persistent hepatic cytolysis, we found that 10 (50%) had steatosis, 9 (45%) had fibrosis and 7 (35%) had steatohepatitis, according to the results of SteatoTest, FibroTest and NashTest, respectively. The FibroTest did not seem associated with the usual metabolic risk factors, suggesting other mechanisms leading to hepatic fibrosis in FMF patients.

A quarter of our FMF patients (5/20), who were included because of previously elevated transaminase levels, no longer had elevated transaminase levels at inclusion. In these patients, FibroMax results were normal. Therefore, in case of elevated transaminase levels in FMF, biological monitoring should be repeated before further explorations.

Even if a part of the high prevalence of liver involvement in our cohort was probably due to a selection bias since our patients were selected based on elevated transaminase levels, half (10/20) of our FMF patients had steatosis according to the results of the SteatoTest. This prevalence is higher than reported in the general population, evaluated between 20 and 30%^17,18^. The prevalence of steato-hepatitis as suggested by the results of the NashTest was 7/20 (35%), whereas the prevalence in the general population is about 15%, but very few studies have explored this issue because liver biopsy is mandatory for the diagnosis^19,20^. Similarly, the prevalence of liver fibrosis, as suggested by the results of the FibroTest, was 9/20 (45%) in our population, higher than the prevalence in the general population estimated between 5 and 15%^18,21,22^.

On the other hand, the prevalence of metabolic syndrome, a major risk factor for NAFLD, in our cohort (5/20, 25%) is similar to the prevalence previously reported in the French general population (21%)^23^. Therefore, our results suggest another pathway for the occurrence of liver impairment in FMF patients, as it has been hypothesized before^4^. Of note, interleukin-1, an upregulated cytokine in FMF, promotes hepatic steatosis and liver inflammation^24^, and may suggest the role of uncontrolled inflammation in the development of NAFLD. Thus, 18/20 (90%) of our patients were homozygous for the M694V *MEFV* mutation, which is known to be associated with more severe forms of FMF^25,26^, and has been previously found more frequently in patients with liver impairment^2^. On the contrary, colchicine did not seem to be associated with FibroMax results. Liver damage has been described in colchicine intoxication with more than 5 mg/day, a higher dose than prescribed in FMF^27^. In their study, Tweezer-Zaks et al.^28^ reported that the mean cumulative colchicine dose before the first appearance of liver abnormalities in 9 FMF patients with cryptogenic cirrhosis was 8.2 ± 6.3 g, which is equivalent to ten years of treatment with a daily dose of 2 mg, as compared to more than 2000 patients in their cohort with a similar or higher cumulative dose of colchicine but without any liver abnormality. In addition, no significant increased risk of liver toxicity was found in a recent meta-analysis of randomized clinical trials^29^. Colchicine is also an antifibrotic drug, although its efficacy in liver fibrosis and cirrhosis remains to be proven^30^. Therefore, we agree on the latest EULAR guidelines regarding cytolysis in FMF patients stating that patients with elevated liver enzymes should be investigated for causes other than an adverse effect of colchicine^31^.

Our study has several limits. We have a small number of patients included, but a large work-up was performed in each patient to exclude other cause of liver abnormalities. To our knowledge, this is the first study assessing FMF patients with elevated transaminase levels with FibroMax that combines different non-invasive tests to evaluate liver steatosis, necrosis, inflammation and fibrosis, and liver ultrasound, which is the currently recommended screening method for NAFLD in at-risk patients^20^. However, non-invasive methods for assessment of liver involvement have several limitations, including different diagnostic performances according to the studied population and FibroMax has not been validated in FMF patients, and we did not compare these results to a liver biopsy^32–34^. In addition, it would be very informative to include a control group consisting of FMF patients with normal liver enzymes. However, all included patients who no longer have elevated transaminase levels had normal FibroMax results.

## Conclusion

In adult FMF patients with persistent hepatic cytolysis, NAFLD is the first cause to consider even in the absence of usual metabolic risk factors, suggesting other mechanisms leading to NAFLD in these patients. As no significant association was found between the cumulated dose of colchicine and FibroMax, colchicine did not seem to be involved in this disorder. Therefore, we believe that the reduction of colchicine dosage, as recommended by the latest EULAR guidelines regarding FMF in case of significant hepatic cytolysis^31^, should be as short as possible and the dosage reincreased if there is no improvement or if another cause is found. FMF should probably be considered as a disease at risk of developing NAFLD. We think that transaminase levels should be regularly checked in FMF patients and, in case of persistent elevation, steatosis and/or fibrosis biomarkers and a liver ultrasound should be performed.

## Supplementary Information

Supplementary Information.

## Data Availability

The datasets generated during and/or analyzed during the current study are available from the corresponding author on reasonable request.
