# Supplementary material for: Role of non-invasive methods in detecting liver impairment in familial Mediterranean fever adult patients with persistent hepatic cytolysis
Source: Sci Rep. 2022 Oct 5;12:16644. doi: 10.1038/s41598-022-17358-x (PMC9534929; doi:10.1038/s41598-022-17358-x)
Supplement: Supplementary file 1 — Supplementary Information. [file 41598_2022_17358_MOESM1_ESM.docx]

**Supplementary Table 1.** Clinical and biological characteristics of the included familial Mediterranean fever patients according to the results of the SteatoTest.

|  | No steatosis (score ≤0.38) (n=10) | Minimal to significant steatosis (0.38 < score ≤0.69) (n=6) | Severe steatosis (score >0.69) (n=4) | p-value | q-value |
| --- | --- | --- | --- | --- | --- |
| **Clinical characteristics** |  |  |  |  |  |
| Number of metabolic syndrome criteria | 1 [0.25-1.75] | 1 [1-1.75] | 4 [3.5-4] | **0.03** | **0.06** |
| Age (years) | 32 [24.5-55.25] | 51 [34.75-65] | 56 [49.25-59.5] | 0.24 | 0.33 |
| BMI (kg/m²) | 23.2 [22.35-24.07] | 22.9 [21.7-24.1] | 30.8 [29.82-31.55] | **0.02** | **0.05** |
| Waist circumference (cm) | 72 [71-77] (n=9) | 86 [78.75-91] | 104 [100.75-107.5] | **0.02** | **0.06** |
| Cumulative dose of colchicine (g) | 14.2 [8.32-20.3] | 19 [14.3-23.47] | 13.9 [7.95-22.25] | 0.55 | 0.68 |
|  |  |  |  |  |  |
| **Biological characteristics** |  |  |  |  |  |
| Creatininemia (µmol/L) | 63 [57.75-117.75] | 79 [75.25-82] | 73 [56.25-86.25] | 0.74 | 0.77 |
| C-reactive protein (mg/L) | 2.11 [0.73-3.94] | 2.82 [1.52-3.32] | 4.48 [3.13-8.12] | 0.63 | 0.69 |
| Serum Amyloid A (mg/L) | 0 [0-6.85] | 0 [0-9.9] | 0 [0-2.17] | 0.85 | 0.85 |
| Fasting glycemia (mmol/L) | 4.85 [4.64-5.17] | 4.86 [4.62-4.99] | 6.08 [5.48-6.61] | 0.06 | 0.11 |
| Glycated hemoglobin (%) | 5.15 [5.02-5.3] | 5.05 [5-5.17] | 5.75 [5.55-6.05] | **0.03** | **0.06** |
| [Aspartate aminotransferase](https://www.linguee.fr/anglais-francais/traduction/aspartate+aminotransferase.html) (UI/L) | 30.5 [25.5-32.75] | 44.5 [34.75-49.75] | 56.5 [45.75-68.75] | **0.02** | **0.05** |
| Alanine aminotransferase (UI/L) | 37 [33.25-53] | 56.5 [49.25-63.75] | 80 [67-107] | **0.02** | **0.05** |
| Gamma-glutamyl-transpeptidase (UI/L) | 20 [13-28] | 41 [32.75-79.25] | 88 [84.5-90] | **<0.01** | **0.03** |
| Total bilirubin (µmol/L) | 7.5 [7-9.5] | 7.5 [7-11] | 12.5 [11-14] | 0.19 | 0.28 |
| Alkaline phosphatase (UI/L) | 65.5 [62.25-75] | 96 [90.75-111.75] | 87 [78.75-102] | **0.02** | **0.04** |
| High-density lipoprotein cholesterol (mmol/L) | 0.91 [0.87-1.34] | 0.92 [0.84-1.28] | 0.87 [0.69-1.01] | 0.59 | 0.68 |
| Low-density lipoprotein cholesterol (mmol/L) | 2.12 [1.49-2.83] | 2.63 [2.22-3.60] | 2.71 [2.26-3.07] | 0.46 | 0.61 |
| Triglycerides (mmol/L) | 0.9 [0.85-1.21] | 1.23 [0.98-1.35] | 2.3 [2.07-4.03] | **<0.01** | **0.05** |
| Insulin (pmol/L) | 60.65 [57.95-71.4] | 60.65 [42.3-100.45] | 123.45 [113.4-130.27] | 0.048 | 0.10 |
|  |  |  |  |  |  |
| **FibroMax** |  |  |  |  |  |
| FibroTest | 0.20 [0.13-0.25] | 0.38 [0.18-0.42] | 0.61 [0.49-0.66] | 0.12 | 0.19 |
| NashTest | 0.25 [0.25-0.25] | 0.38 [0.25-0.5] | 0.5 [0.5-0.56] | **<0.01** | **0.02** |

Values are displayed as median [quartile 1 – quartile 3]. Significant values (i.e., p-value <0.05 and q-value <0.1) are displayed in bold.

**Supplementary Table 2.** Clinical and biological characteristics of the included familial Mediterranean fever patients according to the results of the FibroTest.

|  | No fibrosis (score ≤0.27) (n=11) | Minimal to moderate fibrosis (0.27 < score ≤0.58) (n=5) | Advanced to severe fibrosis (score >0.58) (n=4) | p-value | q-value |
| --- | --- | --- | --- | --- | --- |
| **Clinical characteristics** |  |  |  |  |  |
| Number of metabolic syndrome criteria | 1 [0.5-1.5] | 1 [1-2] | 3.5 [2.75-4] | 0.04 | 0.11 |
| Age (years) | 31 [27.5-45] | 53 [46-56] | 62.5 [56-69.25] | 0.047 | 0.11 |
| BMI (kg/m²) | 23 [21.85-24.85] | 23.4 [22.3-23.5] | 29.25 [26.7-31.55] | 0.045 | 0.12 |
| Waist circumference (cm) | 72.5 [71.25-82.5] (n=10) | 77 [77-88] | 105 [100.75-109] | 0.03 | 0.23 |
| Cumulative dose of colchicine (g) | 14.6 [8.95-18.6] | 23.5 [23.4-25.5] | 11.5 [7.95-18.65] | 0.13 | 0.20 |
|  |  |  |  |  |  |
| **Biological characteristics** |  |  |  |  |  |
| Creatininemia (µmol/L) | 61 [58.5-84] | 75 [74-79] | 82 [69.75-86.25] | 0.61 | 0.64 |
| C-reactive protein (mg/L) | 3.41 [0.82-4.02] | 1.66 [1.17-2.57] | 11.22 [3.94-22.17] | 0.32 | 0.37 |
| Serum Amyloid A (mg/L) | 0 [0-10.1] | 0 [0-0] | 4.35 [0-9.97] | 0.20 | 0.28 |
| Fasting glycemia (mmol/L) | 4.66 [4.45-4.96] | 5 [4.96-5.47] | 6.07 [5.47-6.61] | 0.02 | 0.33 |
| Glycated hemoglobin (%) | 5.2 [5.1-5.3] | 5 [5-5] | 5.75 [5.52-6.05] | 0.04 | 0.17 |
| [Aspartate aminotransferase](https://www.linguee.fr/anglais-francais/traduction/aspartate+aminotransferase.html) (UI/L) | 31 [26.5-33.5] | 43 [33-51] | 47 [44.25-52.25] | 0.10 | 0.18 |
| Alanine aminotransferase (UI/L) | 40 [33.5-58] | 60 [53-64] | 66 [59.25-74] | 0.16 | 0.23 |
| Gamma-glutamyl-transpeptidase (UI/L) | 28 [14.5-30] | 30 [24-41] | 90 [84.5-93] | 0.03 | 0.18 |
| Total bilirubin (µmol/L) | 7 [7-9] | 8 [7-12] | 15 [11.75-17] | 0.08 | 0.16 |
| Alkaline phosphatase (UI/L) | 77 [65-93.5] | 78 [64-90] | 105 [89.25-118.5] | 0.27 | 0.36 |
| High-density lipoprotein cholesterol (mmol/L) | 0.9 [0.84-1.23] | 0.86 [0.83-0.96] | 1.03 [0.875-1.15] | 0.94 | 0.94 |
| Low-density lipoprotein cholesterol (mmol/L) | 2.1 [1.82-2.75] | 2.6 [2.27-2.65] | 3.34 [2.42-3.88] | 0.58 | 0.64 |
| Triglyceride (mmol/L) | 1.21 [0.85-1.34] | 1.12 [0.94-1.21] | 2.28 [1.69-4.03] | 0.32 | 0.39 |
| Insulin (pmol/L) | 61 [58.5-103.3] | 53.1 [38.7-63.9] | 115.15 [109.57-121.65] | 0.04 | 0.15 |
|  |  |  |  |  |  |
| **FibroMax** |  |  |  |  |  |
| SteatoTest | 0.24 [0.20-0.30] | 0.38 [0.27-0.5] | 0.77 [0.63-0.83] | 0.04 | 0.12 |
| NashTest | 0.25 [0.25-0.25] | 0.25 [0.25-0.5] | 0.5 [0.44-0.56] | 0.10 | 0.17 |

Values are displayed as median [quartile 1 – quartile 3]. Significant values (i.e., p-value <0.05 and q-value <0.1) are displayed in bold.

**Supplementary Table 3.** Clinical and biological characteristics of the included familial Mediterranean fever patients according to the results of the NashTest.

|  | No NASH (score=0.25) (n=13) | Possible NASH or NASH (score>0.25) (n=7) | p-value | q-value |
| --- | --- | --- | --- | --- |
| **Clinical characteristics** |  |  |  |  |
| Number of metabolic syndrome criteria | 1 [1-2] | 2 [1-4] | 0.15 | 0.32 |
| Age (years) | 34 [26-56] | 56 [38.5-62] | 0.29 | 0.43 |
| BMI (kg/m²) | 23 [21.5-24.1] | 28.7 [25.5-30.8] | **0.02** | **0.07** |
| Waist circumference (cm) | 75 [71.75-79.5] (n=12) | 97 [90-104] | 0.05 | 0.13 |
| Cumulative dose of colchicine (g) | 14.2 [10.2-21] | 19 [11.7-23.45] | 0.54 | 0.67 |
|  |  |  |  |  |
| **Biological characteristics** |  |  |  |  |
| Creatininemia (µmol/L) | 65 [60-83] | 79 [67.5-85] | 0.76 | 0.79 |
| C-reactive protein (mg/L) | 2.59 [1-4.11] | 3.41 [1.11-4.47] | 0.88 | 0.88 |
| Serum Amyloid A (mg/L) | 0 [0-7] | 0 [0-4.35] | 0.65 | 0.71 |
| Fasting glycemia (mmol/L) | 4.76 [4.64-5.24] | 5.23 [4.98-6.07] | 0.21 | 0.34 |
| Glycated hemoglobin (%) | 5.2 [5-5.3] | 5.4 [5.05-5.75] | 0.36 | 0.50 |
| [Aspartate aminotransferase](https://www.linguee.fr/anglais-francais/traduction/aspartate+aminotransferase.html) (UI/L) | 32 [27-35] | 48 [41-58] | 0.04 | 0.13 |
| Alanine aminotransferase (UI/L) | 45 [34-60] | 68 [57-87] | **0.02** | **0.07** |
| Gamma-glutamyl-transpeptidase (UI/L) | 28 [13-29] | 77 [41-88] | **0.02** | **0.09** |
| Total bilirubin (µmol/L) | 7 [7-10] | 12 [8-12.5] | 0.14 | 0.33 |
| Alkaline phosphatase (UI/L) | 69 [63-93] | 90 [79-110] | 0.09 | 0.22 |
| High-density lipoprotein cholesterol (mmol/L) | 0.92 [0.86-1.39] | 0.88 [0.79-1.03] | 0.54 | 0.63 |
| Low-density lipoprotein cholesterol (mmol/L) | 2.27 [1.78-2.91] | 2.6 [2.23-3.34] | 0.53 | 0.70 |
| Triglyceride (mmol/L) | 0.9 [0.84-1.21] | 2.04 [1.36-2.3] | **<0.01** | **0.04** |
| Insulin (pmol/L) | 61 [57.4-73.9] | 111.2 [76.8-123.45] | 0.19 | 0.32 |
|  |  |  |  |  |
| **FibroMax** |  |  |  |  |
| SteatoTest | 0.24 [0.21-0.3] | 0.72 [0.52-0.83] | **<0.01** | **0.02** |
| FibroTest | 0.23 [0.13-0.34] | 0.43 [0.26-0.61] | 0.17 | 0.32 |

Values are displayed as median [quartile 1 – quartile 3]. Significant values (i.e., p-value <0.05 and q-value <0.1) are displayed in bold.
